# Partial or focal brachytherapy for prostate cancer: a systematic review and meta-analysis

**DOI:** 10.1093/bjr/tqae254

**Published:** 2024-12-19

**Authors:** Enrique Gutiérrez-Valencia, Inmaculada Navarro-Domenech, Kailee Zhou, Marc Barcelona, Rouhi Fazelzad, Matthew Ramotar, Irving Sanchez, Victor Ruiz, Robert Weersink, Rachel Glicksman, Joelle Helou, Alejandro Berlin, Peter Chung, Ronald Chow, Srinivas Raman

**Affiliations:** Princess Margaret Cancer Centre, University Health Network, Temerty Faculty of Medicine, University of Toronto, Toronto, ON, M5G 2C4, Canada; Princess Margaret Cancer Centre, University Health Network, Temerty Faculty of Medicine, University of Toronto, Toronto, ON, M5G 2C4, Canada; Princess Margaret Cancer Centre, University Health Network, Temerty Faculty of Medicine, University of Toronto, Toronto, ON, M5G 2C4, Canada; Princess Margaret Cancer Centre, University Health Network, Temerty Faculty of Medicine, University of Toronto, Toronto, ON, M5G 2C4, Canada; Princess Margaret Cancer Centre, University Health Network, Temerty Faculty of Medicine, University of Toronto, Toronto, ON, M5G 2C4, Canada; Princess Margaret Cancer Centre, University Health Network, Temerty Faculty of Medicine, University of Toronto, Toronto, ON, M5G 2C4, Canada; Department of Radiation Oncology, Western National Medical Center, Mexican Institute of Social Security, UdG School of Medicine, University of Guadalajara, Guadalajara, 44340, Mexico; Department of Radiation Oncology, Western National Medical Center, Mexican Institute of Social Security, UdG School of Medicine, University of Guadalajara, Guadalajara, 44340, Mexico; Princess Margaret Cancer Centre, University Health Network, Temerty Faculty of Medicine, University of Toronto, Toronto, ON, M5G 2C4, Canada; Princess Margaret Cancer Centre, University Health Network, Temerty Faculty of Medicine, University of Toronto, Toronto, ON, M5G 2C4, Canada; London Regional Cancer Program, London Health Sciences Centre, Schulich School of Medicine & Dentistry, University of Western Ontario, London, ON, N6A 5W9, Canada; Princess Margaret Cancer Centre, University Health Network, Temerty Faculty of Medicine, University of Toronto, Toronto, ON, M5G 2C4, Canada; Princess Margaret Cancer Centre, University Health Network, Temerty Faculty of Medicine, University of Toronto, Toronto, ON, M5G 2C4, Canada; Princess Margaret Cancer Centre, University Health Network, Temerty Faculty of Medicine, University of Toronto, Toronto, ON, M5G 2C4, Canada; Princess Margaret Cancer Centre, University Health Network, Temerty Faculty of Medicine, University of Toronto, Toronto, ON, M5G 2C4, Canada

**Keywords:** focal prostate brachytherapy, focal salvage brachytherapy, HDR and LDR focal brachytherapy, partial or focal prostate brachytherapy

## Abstract

**Objectives:**

Recent advances in image-guided brachytherapy have allowed for treatment volume reduction in the treatment of prostate cancer, with the aim to optimize disease control and reduce toxicities. This systematic review reports on the efficacy and safety of focal brachytherapy for treatment of patients with localized prostate cancer.

**Methods:**

Medline, Embase, Web of Science, and Cochrane were searched from inception to July 2023. Studies were included if they reported on focal brachytherapy, and described either dosimetry or clinical outcomes in the monotherapy or salvage setting. Meta-analysis was conducted to estimate biochemical control (BC) at 12-60 months. The review protocol was registered on PROSPERO (CRD42022320921).

**Results:**

Twenty-six studies reporting on 1492 patients were included in this review. Fourteen studies reported on monotherapy, 10 on salvage, and two on boost. The majority of studies used MRI and/or biopsy or PET for target identification, and MRI fusion and transrectal ultrasound (TRUS) for image guidance technique. BC for monotherapy was 97% (95% CI: 86%-99%) at 24 months and 82% (95% CI: 65%-92%) at 60 months. BC for salvage was 67% (95% CI: 62%-72%) at 24 months and 35% (95% CI: 17%-58%) at 60 months. Low rates of toxicity were reported across studies.

**Conclusions:**

Focal brachytherapy has promising efficacy and safety profiles. Future studies may compare focal brachytherapy to whole-gland treatments, to investigate relative efficacy and safety.

**Advances in knowledge:**

In well-selected patients, partial or focal brachytherapy represents an evidence-based option with acceptable BC rates and a favourable toxicity profile.

## Introduction

Prostate cancer is the most common cancer among men, and second leading cause of cancer death among men in the United States. It is estimated that one in six men will develop prostate cancer during their lifetime and approximately one in 35 men will die from prostate cancer.^1^ Curative-intent treatment modalities include radical prostatectomy, external beam radiation therapy, and brachytherapy, either as monotherapy or as a boost following external beam radiotherapy (EBRT).^2^^,^^3^ Brachytherapy, relative to external beam radiotherapy, has been associated with increased rates of tumour control, but also increased urinary toxicity. To mitigate the side effects of whole-gland brachytherapy, focal brachytherapy has gained recent interest in its utility as monotherapy or after external beam radiotherapy as a boost, or as a salvage strategy.^4^

**Figure 1.**
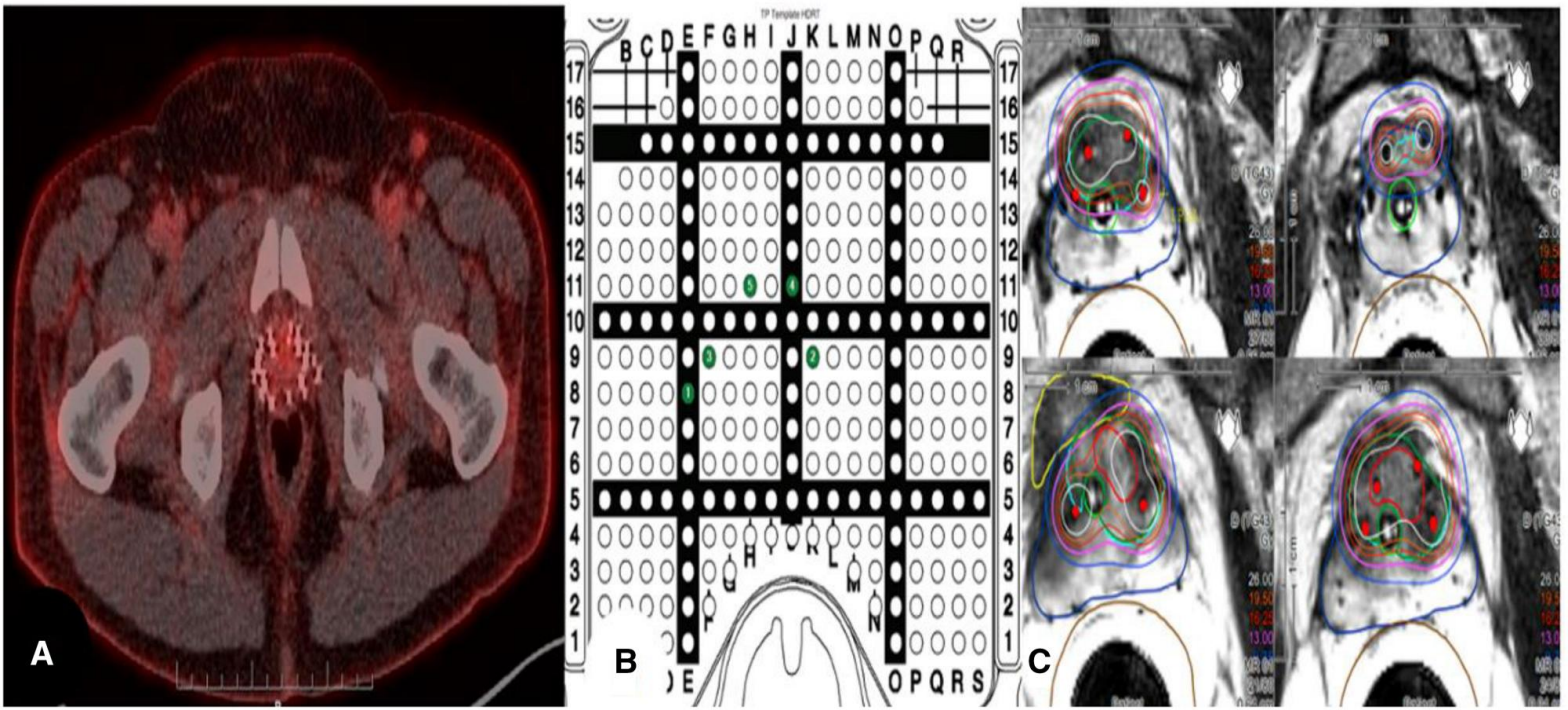
Salvage focal salvage HDR-BT in a patient previously treated with LDR-BT. (A) A prostate-specific membrane antigen positron emission tomography (PSMA PET) scan with an area of uptake at the anterior portion of the prostate apex; the biopsy of the lesion confirmed local recurrence. (B) The location of the catheter insertion by MRI guidance during focal salvage HDR-BT, and (C) Contoured in light blue PTV. Isodose lines: purple 13 Gy, orange 16.25 Gy, yellow 19.5 Gy.

In the monotherapy setting, where patients have favourable disease characteristics and limited prostate involvement, focal brachytherapy can be employed as a de-escalation strategy to achieve durable disease control. Additionally, since local recurrences usually occur at the same site as the dominant intraprostatic lesion (DIL) in patients treated with radiation therapy to the whole gland,^5^ focal brachytherapy also seems to be a rational salvage strategy in which reirradiation (Figure 1) represents a challenging scenario due to the risk of treatment-associated toxicities.

Technological advances such as multi-parametric MRI and multimodal fusion have enabled partial prostate brachytherapy to be applied more broadly.^4^ In the external beam radiotherapy literature, there is a strong body of evidence including data from randomized trials that DIL boost increases the therapeutic ratio of radiotherapy delivery. In brachytherapy, there is less evidence to support the utility of partial/focal treatment and data are mostly limited to single institutional series and non-randomized trials. To address the heterogeneity in data, and synthesize the published evidence, we report a systematic review and meta-analysis summarizing biochemical control (BC) and toxicity rates in patients with localized prostate cancer treated with focal or partial brachytherapy as monotherapy or salvage modality.

## Methods

This systematic review was conducted according to the Cochrane Handbook for Systematic Reviews of Interventions^6^ and reported based on the Preferred Reporting Items for Systemic Reviews and Meta-Analyses (PRISMA) 2020 and PRISMA 2020 for Abstracts Checklist.^7^ The protocol was registered with the International Prospective Register of Systematic Reviews (PROSPERO) (ID CRD42022320921).

### Search strategy

A comprehensive literature search was performed in Medline, Embase, Cochrane Central Register of Controlled Trials, Cochrane Database of Systematic Reviews, and Web of Science from the inception of each database to July 2023. Search strategy was developed to identify studies reporting on patients with prostate cancer and brachytherapy. The search strategy included a combination of controlled vocabulary terms and text words, adapting the database-specific search syntax. The search was limited to human studies and articles published in English, excluding book, conferences, and preprints (Supplementary Material 1A and B).

### Eligibility

In level 1 screening, articles were eligible for further screening if they reported on focal or partial brachytherapy in their title and abstract. Focal brachytherapy was defined as treatment delivered to intra-prostate disease identified on imaging, and partial brachytherapy referred to treatment delivered to distinct anatomical zones, such as peripheral zone or hemigland treatment. In level 2 screening, articles were included in this review if they reported on >10 patients in their full text.

All screening was done independently, each article was read by each reviewer (E.G., I.M., K.Z.). All decisions required consensus by review authors. When there were discrepancies that could not be resolved by discussion and subsequent consensus, a senior author (S.R.) was involved in the discussion.

### Data extraction

For each article included in this review, patient characteristics were extracted, including age, Gleason score, prostatic-specific antigen, and risk classification. Dosimetry metrics including brachytherapy type (HDR or LDR), prescribed dose, number of fractions, imaging techniques, and toxicity as measured by the Common Terminology Criteria for Adverse Events (CTCAE) were recorded. Studies were categorized into either using focal brachytherapy as monotherapy or salvage.

BC at predetermined timepoints of 12, 18, 24, 36, 48, and 60 months were extracted from the full-text and/or figures for monotherapy and salvage studies when reported.

### Meta-analysis

Meta-analysis was conducted to summarize proportion of patients achieving BC at the pre-specified timepoints. For the purposes of this study, BC was defined as the absence of biochemical failure (BF), and definitions of BF were collected for all the studies. Common and random effects models were used to generate summary proportions with accompanying 95% confidence intervals. To visually depict the results, forest plots were generated. Heterogeneity was quantified using the I2 statistic, where values equal to or exceeding 50% indicated substantial heterogeneity. The random effects model was used as the summary estimate if *I*^2^ > 50%. Type I error was set at 0.05. All statistical analyses were conducted using R 4.3.0 (R Foundation for Statistical Computing, Vienna, Austria). Furthermore, study quality and the possible risk of bias were assessed for each study using the ROBINS-I tool.

## Results

**Figure 2.**
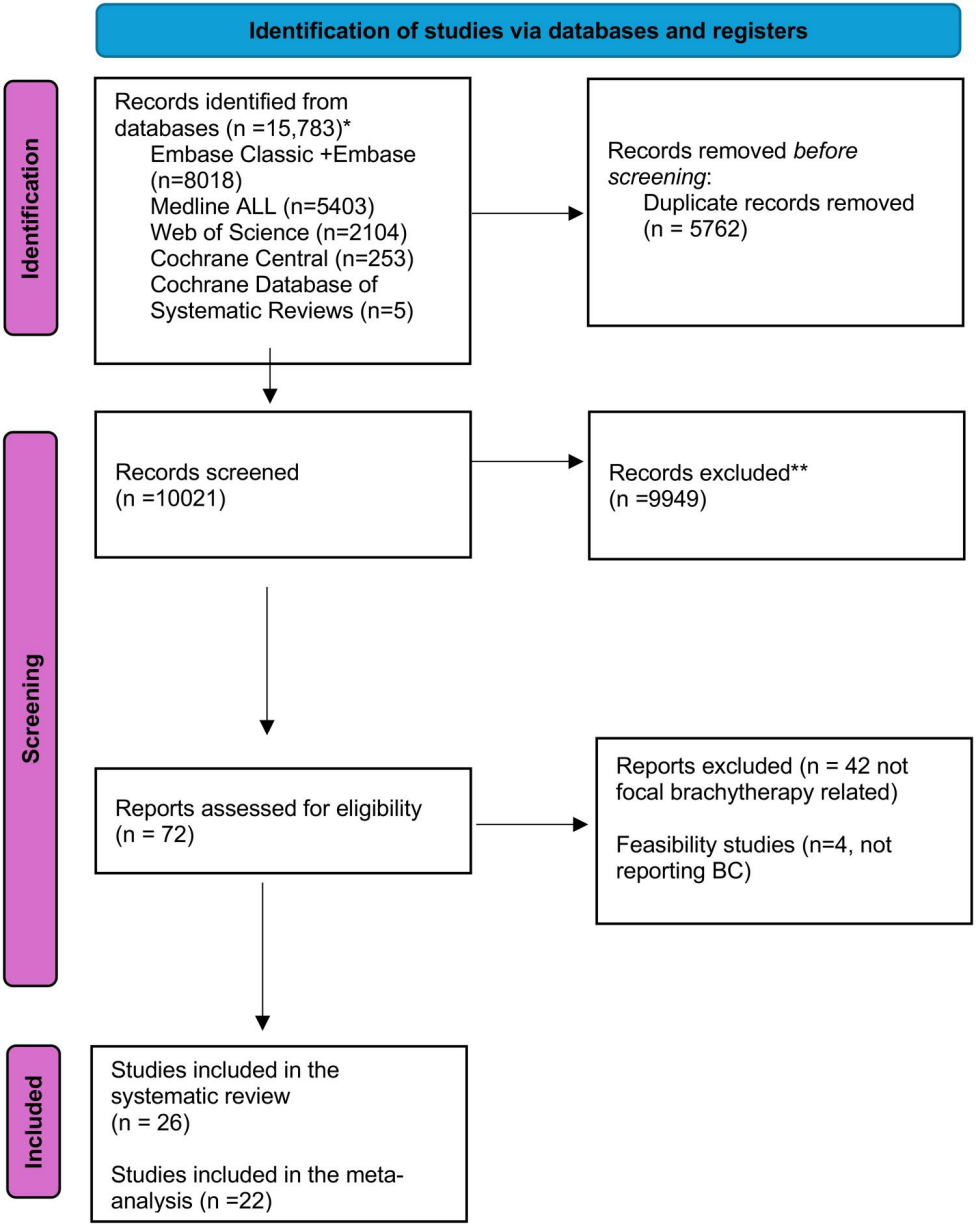
Study flow chart. *Consider, if feasible to do so, reporting the number of records identified from each database or register searched (rather than the total number across all databases/registers). **If automation tools were used, indicate how many records were excluded by a human and how many were excluded by automation tools.

A total of 15 783 studies were identified. After removing duplicates and excluding studies that did not meet the inclusion criteria, 26 studies were included in the systematic review. Four studies were excluded from the meta-analysis: two involved brachytherapy boost, one did not report BC at a minimum of six months, and one included whole-gland treatment with a focal arm of fewer than 10 patients. Ultimately, 22 studies were included in the meta-analysis for BC (Figure 2), BF was determined in 18 studies as the Phoenix criteria (PSA nadir + 2.0 ng/mL),^8^ one study used Phoenix criteria or the American Society for Therapeutic Radiology and Oncology (ASTRO) consensus definition of three consecutive rising PSA measurements,^9^ one study used Phoenix criteria plus a PSA velocity ≥0.75 ng/mL per year between the nadir and the value before salvage and two studies did not specify the definition of BF. The definitions used in each study are provided in Supplementary Material 2A/B.

Fourteen studies were included as F-BT monotherapy. Thirteen of these studies reported BC between 6 and 60 months.^10–27^ Ten studies were included on salvage F-BT^4^^,^^28–37^ and two studies on F-BT boost.^38^^,^^39^ There were a total of 1492 patients with Pca treated with F-BT; 747 as monotherapy, 600 salvage, and 145 F-BT boost. High dose-rate brachytherapy (HDR-BT) and low dose-rate brachytherapy (LDR-BT) were employed in 13 studies each, respectively. HDR-BT and LDR-BT were used in four and 10 trials for F-BT as monotherapy and seven and three for salvage, respectively. Twenty studies were prospective, and all reported baseline PSA levels, age, Gleason score or grade group (with the majority having a Gleason score <7), technique used (LDR or HDR), and number of fractions for the HDR population. The site of recurrence was reported in 17 out of 26 studies. Individual study characteristics are reported in Tables 1–3.

**Table 1.** Characteristics of the monotherapy studies included.

Study	Type	Median age	Number of patients	Type	Dose (Gy)	Fractions	Target identification	Imaging technique	Target definition	PSA	Gleason	D90 DIL	V100 DIL	Whole organ dose	Toxicity	QoL	BF and Site of recurrence	LC (biochemical)
Laing et al 2016	Prospective	66	22	LDR	145	1	MRI + biopsy	TRUS	Hemigland	≤15 ng/mL	≤7	153.8 Gy	93.1%	54.7 Gy	NR	NR	NR	
Graff et al 2018	Prospective	63.4	17	LDR	160	1	MRI + biopsy	MRI + TRUS	GTV + 2 mm	2.7-11.4 ng/mL	≤6	NR	95%	14.5%	Negligible	Not affected.	Local 1 (out of field)	100% at 1 years
King et al 2018	Retrospective	64.1	354	LDR	137	1	MRI	MRI	Peripheral zone	ng/mL ≤4 *n* = 106 (30%) 4-10 ng/mL *n* = 232 (66%) >10 *n* = 16 (5%)	3 + 3 = 6 *n*294 (83%) 3 + 4 = 7 *n*55 (16%) 4 + 3 = 7 *n*4 (1%) 4 + 4 = 8 *n*1 (0)	NR	NR	NR	NR	NR	BF 33.8 at 4.8 years Local 44 (19 out of field)	66.2% at 4.8 years
Maenhout et al 2018	Prospective	68	30	HDR	19	1	MRI	MRI	GTV + 5 mm	≤10 ng/mL	3 + 3 = 6 *n*16 (53%) 3 + 4 = 7 *n*12 (40%) 4 + 3 = 7 *n*2 (7%)	20.8 Gy	NR	NR	3.33% G3 GU	No statistically significant deterioration in QoL in all questionnaires	BF 16.6% Bone 2, 1 local (biopsy proven), 2 unidentified	83.33% at 24 moths
Langley et al 2019	Prospective	65.6	30 hemigland	LDR	145	1	MRI + biopsy	MRI	Hemigland	≤15 ng/mL Mean 6.7	3 + 3 = 6 *n*5 (17%) 3 + 4 = 7 *n*21 (70%) 4 + 3 = 7 *n*4 (13%)	153Gy	92.7%	NR	No differences bt HG & WG	Moderate improvement from pre-treatment symptoms in HG patients (IPSS, QoL, IIEF5)	Local 1	97% at 55 months
Peters et al 2019	Prospective	71	30	HDR	19	1	MRI + biopsy	MRI	GTV + 5 mm	≤10 ng/mL Median 7.3 ng/mL (5.2-8.1)	3 + 3 = 6 *n*16 (53.3%) 3 + 4 = 7 *n*12 (40%) 4 + 3 = 7 *n*2 (6.7%)	20.8 Gy	NR	NR	0% GI/GU G3	IPSS deteriorated temporarily Long-term clinically relevant QoL deterioration was seen in sexual activity and tiredness, emotional and cognitive functioning improved	BF 30% at 4 years Local 9 (seven out of field)	70% at 4 years
Kim et al 2020	Retrospective	65.5	30	LDR	145	1	MRI + biopsy	TRUS	Focal BT: cancer foci Partial BT: cancer foci + remainder of prostate	7.6 (5.5-10.3) ng/mL	6 *n*17 (56.7%) 7 *n*11 (36.7%) 8 *n*2 (6.7%)	NR	NR	NR	No G3 GI, 16.6% GU	NR	BF 8.2 at 3 years	91.8% at 3 years
Kunogi et al 2020	Prospective	73	19	LDR	145	1	MRI + biopsy	MRI	GTV + 5 mm	Median PSA 7.3 (4.7-9.8)	3 + 3 = 6 *n*8 (42%) 3 + 4 = 7 *n*10 (53%) 4 + 3 = 7 *n*1 (5%)	222Gy	NR	34.6%	15% G2 GU at 12 months 10.5% G2 GU at 24 months	NR	1 (seminal vesicle)	92.9% at 2 years
Prada et al 2020	Prospective	67	50	HDR	24	1	MRI + biopsy	TRUS	Ultra-focal visible tumour vs hemigland	≤10 ng/mL *n*48 (96%) 10.1-13 (4%)	≤6 *n*31 (62%) 7 *n*19 (38%)	23Gy	90%	D90 4.7 Gy	None	NR	BF 23 % at 3 years Local 7 (five out of field)	77% at 3 years/62% at 5 years
Anderson et al 2021	Retrospective	71	26	LDR	145	1	MRI/PET + biopsy	MRI	GTV + 7 mm	7.3 (3.1 SD)	3 + 3 = 6 *n*1 (3.8%) 3 + 4 = 7 *n*25 (96.2%)	NR	92.3%	31.7%	G2 GU and erectile reported by 29.2% and 45.8% No G ≥ 3 GU or erectile or G ≥ 2 GI toxicities	NR	BF 3.8 at 24 months Local 1 (in-field)	96.2% at 24 months
Saito et al 2021	Prospective		24	LDR	160	1	MRI + biopsy	TRUS	Hemigland	8.0 (6.1-10.1) ng/mL	GG 1 *n*4 (17%) 2 *n*18 (75%) 3 *n*2 (8%)	150.2 Gy	87.4%	NR	NR	The IPSS significantly deteriorated at 3 months and reversed itself afterward. The IIEF5 had no significant decrease	BF 29% at 60 months Local 6 (five out of field)	71% at 60 months
Ta et al 2021	Retrospective	62	39	LDR	145	1	MRI + biopsy	MRI	GTV + 1 cm	7.12 (5.7-7.98) ng/mL	3 + 3 = 6 *n*32 (82.1%) 3 + 4 = 7 *n*7 (17.9%)	182.7 Gy	99.7%	NR	No acute or late GI/GU G3	The 2-month IPSS score showed a significant increase compared to baseline (p = 0.0003), but no statistically significant differences were observed at subsequent time points. GU toxicity remained stable after 6 months	BF 3.2% at 5 years Local 7 (out of field)	96.8% at 5 years
Kamitani et al 2022	Prospective	69	25 (8 focal)	HDR	19	1	MRI + biopsy	MRI	GTV + 5 mm	Median 5.7 (2.36-10.36) ng/mL	3 + 3 = 6 *n*3 3 + 4 = 7 *n*5	D95 116.32%	84.5%	NR	0%	NR	BF 12.5% Local 1 (in & out field)	87.5% at 7.75 months
Matsuoka et al 2022	Prospective	68	51	LDR	160	1	MRI + biopsy	TRUS	GTV + 5 mm	Median 6.9 (5.7-9.2) ng/mL	GG 1 *n*21 (41%) 2 *n*26 (51%) 3 *n*4 (8%)	149.9 Gy	86.3%	NR	GU %	After 3-6 months it was recovered to baseline, and pad-free continence and ejaculation was preserved in higher rate compared to radical prostatectomy	BF 21% at 5 years Local 16 (11 out of field)	79% at 5 years

Abbreviations: D90 DIL = the dose received by 90% of the dominant intraprostatic lesion (DIL); GG = grade group; GI = gastrointestinal; GU = genitourinary; IIEF5 = international index of erectile function 5; IPPS = international prostate symptom score; IQR = interquartile range; MCIC = minimal clinically important change; NR = no reported; QoL = quality of life; SD = standard deviation; TRUS = transrectal ultrasound; V100 DIL = percent volume of DIL that is encompassed by the 100% or greater isodose surface.

**Table 2.** Characteristics of the salvage studies included.

Study	Type	Median age	Number of patients	Type	Dose (Gy)	Fractions	Target identification	Imaging technique	Target definition	PSA	Gleason	D90 DIL	V100 DIL	Whole organ dose	Toxicity	QoL	BF and Site of recurrence	LC (biochemical)
Hsu et al 2012	Prospective	68	15	LDR	144	1	Biopsy	MRI	Focal MRI abnormality. Underdosage areas (CTV)	3.5 (0.9-5.6) ng/mL	6 *n*10 (66.7%) 7 *n*2 (13.3%) 8 n2 (13.3%)	141.8 Gy	89.7 Gy	36.8 Gy	No G 3-4 GI/GU. 13% G1 GI, 33% G2 GU	NR	BF 21.6% Local 2 (in-field)	78.4% at 2 years
Peters et al 2014	Retrospective	69	20	LDR	145	1	MRI + biopsy	MRI	(GTV + expansion up to hemigland	4-10 *n*8 (40%) 10-<20 *n*7 (35%) 20-<50 *n*4 (20% >50 *n*1 (5%) ng/mL	Grade ≤6 *n*12 (60%) Grade 7 *n*6 (30%) Grade 8-10 *n*2 (10%)	200 Gy	100 Gy	44 Gy	5% G3 GU	Only GU decreased after 3 years	BF 30% 3 (NS)	70% at 36 months
Kunogi et al 2016	Prospective	69.16	12	LDR	145	1	Mapping biopsy	TRUS	Positive biopsy area + 3 mm	8.89 (5.03-11.8) ng/mL	3 + 3 = 6 *n*5 3 + 4 = 7 *n*2 4 + 3 = 7 *n*2 4 + 4 = 8 *n*2 NA = 1	200 Gy	NR	86%	No G3, GU 33% @1, 2, &3 years, 25% @4 years GI 8.3% @2 years	NR	BF 22% at 4 years 2 (NS)	78% at 4 years
Maenhout et al 2017	Prospective	58-78	17	HDR	19	1	Biopsy + MRI + PET	MRI	GTV + 5 mm	3.3-28 ng/mL	Gleason 5= *n*2 6= *n*10 7= *n*4 8= *n*1	D95 CTV 18.9 Gy (*average)*	NR	NR	1 pt chronic G3 GU	NR	BF 6% due to distant nodal metastasis	94% at 10 months
Chitmanee et al 2020	Prospective	63	50	HDR	19	1	MRI + PET	MRI	Positive biopsies + MRI abnormalities + 3 mm	≤10 *n*23 (46%) >10 and ≤20 *n* 16 (32%) >20 *n*11 (22%) ng/mL	GG 5 *n*1 (2%) 6 *n*20 (40%) 7 *n*24 (48%) 8 *n*3 (6%) 9 *n*2 (4%)	22.1 Gy	100%	NR	10 % G3 GU	NR	BF 54% at 3 years 13 (2 local)	46% at 3 years
Slevin et al 2020	Retrospective	70	43	HDR	19	1	Biopsy + MRI + PET	TRUS	GTV + 3 mm	Median 10.5 (3.4-178) ng/mL	GG 1 *n*21 (49%) 2 *n*14 (33%) 3 *n*4 (9%) 4 *n*2 (5%) 5 *n*2 (5%)	18.6 Gy	88.5%	NR	G2 acute GU/GI (91%) and (14%). G2 late GU and GI (65%) and (14%). 2% G3 late GU	NR	17 (six local)	41.8% at 3 years
Willigenburg/van Son et al 2021 2020	Prospective	72	150	HDR	19	1	MRI + PET	MRI	GTV + 5 mm	Median 4.88 (2.80-6.80) ng/mL	Gleason at salvage, n (%) 3 + 3 = 6 *n*14 (9.3%) 3 + 4 = 7 *n*27 (18.0%) 4 + 3 = 7 *n*21 (14.0%) 8 *n*6 (4.0%) 9/10 *n*14 (9.3)	19Gy	NR	NR	Acute G2/3: 41%/3% (GU), 5%/0% (GI), 22% and 15% (ED). Chronic G2 GU, ED were seen twice as frequent	NR	61 (NS)	59% @ 32.9 months
Corkum et al 2022	Prospective	72	30	HDR	27	2	MRI + biopsy	MRI	GTV + 3 mm	Median 4.2 (1.3-11.6) ng/mL	Gleason score at salvage, *n* (%) 6 *n*3 (10%) 7 *n*20 (67%) 8-9 *n*7 (23%)	NR	96.5%	NR	No acute G ≥ 3 GU/GI. One temporary late G3 GU, no late G ≥ 3 GI	The sexual domain declined. No Gu changes	17 (three patients developed distant and local failure, two patients showed evidence of local failure on MRI or PSMA PET)	61.8% at 36 months 21% at 60 months
Menard et al 2022	Prospective	71	88 (73 focal)	HDR	22-26	2	MRI + biopsy	TRUS/MRI	GTV + 5 mm	Median 4.2 (1.2, 24) ng/mL	GG 1-3 *n*41 (59%) 4-5 *n*28 (41%)	NR	NR	NR	No attributable G3 events. ibBT associated with a higher rate of grade 2 toxicity	Urinary and bowel QoL returned to baseline 3 months after salvage BT, while sexual function decline persisted	LF 21% at 3 years	49% at 5 years
Rasing et al 2023	Prospective	72	175	HDR	19	1	MRI	TRUS/MRI	GTV + 5 mm	PSA 4.4 (2.5-6.7) ng/mL	Gleason 6 *n*91 (52.0%) 7 *n*64 (36.6%) 8 *n*11 (6.3%) 9/10 *n*6 (3.4%) Unknown *n*3 (1.7%)	NR	NR	NR	NR	NR	84 (44 local-only)	43% at 3 years

Abbreviations: BF = biochemical failure; D90 DIL = the dose received by 90% of the dominant intraprostatic lesion (DIL); GI = gastrointestinal; GU = genitourinary; IIEF5 = international index of erectile function 5; IPPS = international prostate symptom score; MCIC = minimal clinically important change; NR = no reported; QoL = quality of life; TRUS = transrectal ultrasound; V100 DIL = percent volume of DIL that is encompassed by the 100% or greater isodose surface.

**Table 3.** Characteristics of the BOOST studies included.

Study	Type	Median age	Number of patients	Type	Dose (Gy)	Fractions	Target identification	Imaging technique	Target definition	PSA	Gleason	D90 DIL	V100 DIL	Whole organ dose	QoL	BF and site of recurrence	LC (biochemical)
Gomez et al 2016	Prospective	71	15	HDR	15	1	MRI + biopsy	MRI	Visible dominant intra-prostatic nodule on mpMRI	9 (5.1-40) ng/mL	6 *n*2 (12%) 7 (3 + 4) *n*5 (33%) 7 (4 + 3) n3 (20%) 8 n2 (13%) 9 (20%)	142.7 %	100%	98.1% (97.8-99.1)	NR	NR	100% @18 months
Kovacs et al 2016	Prospective	68	130	HDR	30	2	Biopsy	TRUS	Dominant lesions detected by multiparametric TRUS	18.69 (0.75-140)	6.78 (3-9)	6.58 Gy	30.36%	NR	NR	Two local Two distant	97% @4.3 years

Abbreviations: BF = biochemical failure; D90 DIL = the dose received by 90% of the dominant intraprostatic lesion (DIL); GI = gastrointestinal; GU = genitourinary; IIEF5 = international index of erectile function 5; IPPS = international prostate symptom score; MCIC = minimal clinically important change; NR = no reported; QoL = quality of life; TRUS = transrectal ultrasound; V100 DIL = percent volume of DIL that is encompassed by the 100% or greater isodose surface.

The risk of bias assessment for the studies included in the meta-analysis using the ROBINS-I tool indicated that 16 studies (72.8%) had a low risk of bias, and six studies (27.2%) were categorized as having a moderate risk of bias. Detailed assessments and categorization for each study are provided in Supplementary Material 3A and B.

### Target definition and dosimetry

Radiation dosimetry was described in 22 out of 26 manuscripts from 2014 to 2023 (feasibility studies were excluded).^10^^,^^13^^,^^17^^,^^34^ In total, 14 (54%) F-BT studies as monotherapy, 10 (38%) as focal salvage treatment and two (8%) as boost were included. Target definition and identification, image guidance during brachytherapy procedure, and dosimetry values for DIL and whole prostate are reported in Tables 2 and 3.

The majority, 23 (88.4%) studies, were designed using the MRI ± biopsy or PET for target identification. As monotherapy treatment, 10 (71.4%) used LDR-BT with a median dose of 145 Gy (range 137 Gy, 160 Gy), and four (28.6%) HDR-BT with a median dose of 19 Gy in a single fraction (range 19 Gy/1 fraction, 24 Gy/2 fractions). The median D90 to the DIL was 153.4 Gy (range 149.9 Gy, 222 Gy) for LDR-BT and 22 Gy (range 20.8 Gy, 23.25 Gy) for HDR-BT. V100 was 92.5% (range 84.5%, 99.7%) to the DIL and 33.15% (range 14.5%, 38%) to the whole prostate.

For salvage treatment, three (30%) publications used LDR-BT and seven (70%) HDR-BT prescribed to a median dose of 145 Gy (range 144 Gy, 145 Gy) and 19 Gy in a single fraction (range 19 Gy/1 fraction, 27 Gy/2 fractions), respectively. D90 to DIL was 200 Gy (range 141.8 Gy, 200 Gy) and 19 Gy (range 18.6 Gy, 22.1 Gy) for LDR-BT and HDR-BT, respectively. Median V100 to the DIL and to the whole gland was 88.5% (range 62.3%, 100%) and 30.3% (range 25.6%, 86%), respectively.

### Biochemical outcomes

**Figure 3.**
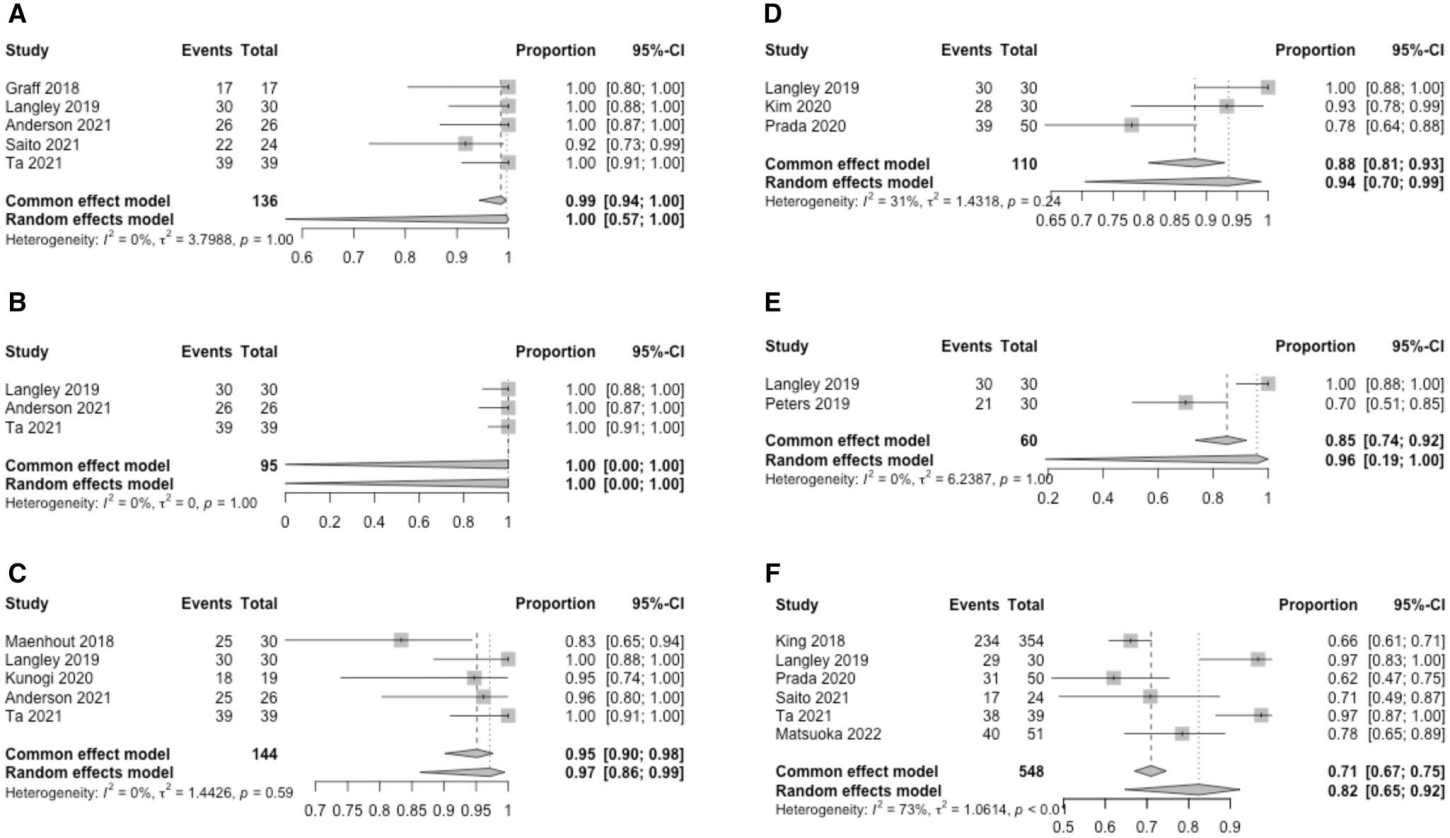
Forest plot displaying the common and random effects model of focal brachytherapy monotherapy studies reporting biochemical control. (A) 12 months. (B) 18 months. (C) 24 months. (D) 36 months. (E) 48 months. (F) 60 months.

**Figure 4.**
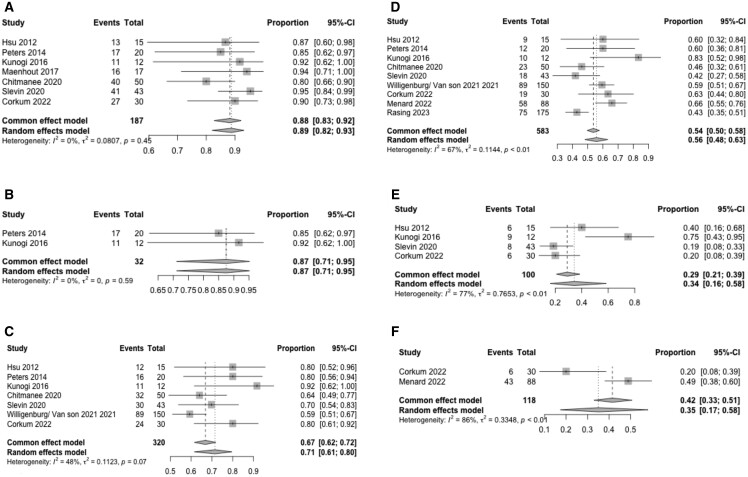
Forest plot displaying the common and random effects model of focal salvage brachytherapy studies reporting biochemical control. (A) 12 months. (B) 18 months. (C) 24 months. (D) 36 months. (E) 48 months. (F) 60 months.

BC common effect model estimate for monotherapy and salvage in five and seven studies indicated at 12 months were 100% (95% CI: 94%-100%) (*P* = 1.00) and 88% (95% CI: 83%-92%) (*P* = .45), respectively; at 24 months for monotherapy and salvage in five and seven studies, were 97% (95% CI: 86%-99%) (*P* = .59) and 67% (95% CI: 62%-72%) (*P =* .07), respectively, and at 36 months in three monotherapy and nine salvage studies BC was 88% (95% CI: 81%-93%) (*P* = .24) and 56% (95% CI: 48%-63%) (*P* ≤ .01), respectively. Monotherapy with F-BT demonstrated sustained BC over time with 82% (95% CI: 65%-92%) (*P* < .01) at 60 months, whereas salvage F-BT was 35% (95% CI: 17%-58%) (*P* ≤ .01) (Figures 3 and 4).

### Toxicity and QoL

Different studies reported varying outcomes in terms of toxicity. In the group receiving F-BT monotherapy, the percentage of reported grade 2 or higher GU toxicity ranged from 0% to 37%, while the percentage of reported grade 2 or higher GI toxicity ranged from 0% to 25%. In the salvage F-BT group, the percentage of reported grade 2 or higher GU toxicity varied from 0% to 65%, while the percentage of reported grade 2 or higher GI toxicity ranged from 0% to 14% (Tables 1-3).

Quality of life (QoL) assessment was reported in 10 (42%) out of 26 manuscripts, seven (29%) for the monotherapy series and three (13%) for salvage brachytherapy. Most of the series did not find a significant change in score in the genitourinary, gastrointestinal, and sexual domain in the first 3-6 months and subsequent recovery. The sexual domain was the most affected in one series^33^ in which 22% newly developed grade 3 erectile dysfunction.

## Discussion

Focal or partial prostate brachytherapy has gained popularity over recent years due to its dosimetric advantages, safety profile, low incidence of side effects, and efficacy reported in multiple studies.^4^^,^^10–37^ In the present study, we review 23 papers that report on dosimetry and/or clinical outcomes of F-BT in the monotherapy and salvage settings, discussing its advantages versus treating the whole gland and the potential superiority regarding toxicity and quality of life.

Most studies investigated prostate brachytherapy in the context of monotherapy. At 60 months, the estimated BC is 78%. These oncologic outcomes are typically lower than typically reported 5-year rates of 90%+ in LDR-BT and HDR-BT whole-gland series. However, most monotherapy studies in our series reported no grade 3 toxicities in their series with only one study reporting 3.3% rate of grade 3 GU toxicity.^16^ This compares favourably to other whole-gland series which have reported higher rates of grade 3+ toxicity.^29^ As expected there appears to be a trade-off between efficacy and toxicity with volume-based treatment intensification. Most studies did not report on detailed patterns of recurrence. However, King et al reported that the main pattern of recurrence was intra-prostatic, with almost 50% of recurrences occurring outside the treated lesion within the prostate.^15^

Salvage therapies after EBRT, such as radical prostatectomy, LDR, or HDR brachytherapy, are often perceived to carry a high risk of GU and GI toxicity, and no single option is clearly superior. Recent studies, like the MASTER meta-analysis, indicate similar efficacy across treatments for 5-year BC, with reirradiation (HDR, LDR) showing lower rates of severe toxicity compared to radical prostatectomy.^40^ In our meta-analysis, 11 studies assessed the role of F-BT in the salvage setting. As demonstrated above, the long-term BC rates were generally lower, reaching only 56% at 3 years, and even lower at 4 years (29%). This is generally lower than the whole-gland outcomes, albeit with less toxicity, as seen in the monotherapy setting. The main patterns of recurrence were intraprostatic and outside the treatment volume.^15^^,^^35^^,^^41^ Differences in BC between monotherapy and salvage partial/focal brachytherapy (PFB) can be explained by the inclusion of most patients with low and intermediate-risk PCa, with the largest study including only very low, low and intermediate-risk PCa patients.^13^ On the other hand, the largest study of salvage F-BT^35^ included 150 patients, of which 59 (39.3%) had high-risk PCa and 65 patients with T3 or greater disease. Therefore, the vastly different patient populations with differing patient and disease characteristics can explain the difference in the BC achieved in these different scenarios.

The techniques used for target definition and image guidance in the systematic review were variable. The majority of studies used multi-parametric MRI and biopsies to define the target. The target was usually the visible tumour (GTV) plus a margin to include microscopic disease. However, some studies treated the hemigland or the peripheral zone only.^5^^,^^12^^,^^18^^,^^24^

Given the variability in target-defining techniques in focal brachytherapy, standardizing the contouring process is crucial for improving reproducibility in future trials. While the ReIGNITE study showed how advanced imaging enhanced contour accuracy in EBRT intraprostatic boosts, our focus on focal brachytherapy underscores the need for tailored, standardized approaches to address its unique challenges.^42^

To the best of our knowledge, there has been no published direct comparison between HDR-BT and LDR-BT techniques in the delivery of focal brachytherapy. HDR-BT does offer several possible advantages, including the use of inverse planning to optimize dose distribution and delivery, accounting for variabilities in needle placement. This advantage may be important as it was noted in the studies that the vast majority of recurrences occurred are marginal^15^^,^^35^^,^^41^ or beyond the implanted peripheral zone in 43% of patients presented with LR,^15^ and HDR-BT may be able to provide better control over the dose distributions until future trials will inform us about the adequate margins and target coverage necessary in this setting. HDR-BT also obviates the need for post-implant dosimetry, increasing efficiency. A possible disadvantage of HDR-BT is that it is optimally delivered in at least two fractions, though, as seen in the studies we have included in this meta-analysis, the vast majority of them employed single fraction HDR-BT with a median dose of around 19 Gy.^43^

The majority of the studies of LDR-BT utilized 145 Gy dosing which is the recommended prescription in the whole-gland monotherapy setting. Almost all the HDR-BT studies included in the meta-analysis utilized only one fraction which has been shown in clinical trials to lead to inferior outcomes.^43^^,^^44^ The two studies reporting a two fraction HDR-BT regime were published by Corkum and Menard colleagues and showed favourable 3- and 5-year BC rates, which are comparable to other salvage techniques such as prostatectomy, HIFU, and cryotherapy. However, there was no comparator arm.^36^ Thus, we encourage more studies utilizing two fraction HDR-BT brachytherapy approach in either the monotherapy or salvage to be conducted so as to determine if this would improve outcomes, provide a comparable safety profile to single fraction HDR-BT, and achieve similar or better outcomes compared to focal LDR-BT.

The use of focal dose escalation has gained significant traction with external beam radiotherapy. The pivotal phase 3 FLAME trial randomized intermediate or high-risk prostate cancer patient to whole-gland radiation alone to a dose of 77 Gy of whole gland with focal simultaneous integrated boost to the DIL up to a dose of 95 Gy showing statistically significant improvement in biochemical disease-free survival.^45^ Prior to publication, Miralbell and colleagues sought to determine if a stereotactic boost to the DIL would be safe and effective in clinically localized prostate cancer patients. They delivered 64-64.4 Gy to the whole gland with a stereotactic boost of two fractions of 5-7 Gy. This resulted in favourable toxicity profiles and a 5-year bDFS of 98% and a 5-year DFS of 100%.^46^

Furthermore, recent clinical trials on stereotactic body radiotherapy (SBRT) using 5-7 fractions have provided valuable insights into the management of localized PCa, demonstrating non-inferiority in BC and similar toxicity profiles when compared to surgery^47^ or conventional EBRT.^48^^,^^49^ Additionally, dose escalation in this context appears feasible using a simultaneous integrated boost technique, achieving up to 40 Gy in five fractions with excellent BC outcomes.^50^ These encouraging results raise the question of whether a direct comparison with partial/focal brachytherapy (PFB) is warranted in a randomized controlled trial in appropriately-selected patients, not only to assess BC outcomes but also to evaluate toxicity, particularly considering the less invasive nature of SBRT and the promising recent findings on toxicity from the PACE-B trial.^49^ As noted in this meta-analysis, there is a paucity in randomized clinical trials on F-BT and more must be done to further elucidate its role and the optimum parameters for its implementation in this promising new paradigm of focal prostate cancer treatment. We are aware of only one ongoing trial comparing focal HDR-BT boost and whole-gland SBRT with whole-gland HDR-BT boost in terms of cancer control, toxicity, and quality of life.^51^

Additionally, there are opportunities to potentially improve outcomes for focal brachytherapy. The consistent use of multi-parametric MRI and saturation biopsies may optimize patient selection and target volume identification. In the salvage setting, the use of increasingly sensitive PSMA PET scans may rule out patients with small-volume regional or distant disease. There is an increasing trend and potential advantages to using HDR-BT brachytherapy for focal treatments, as mentioned above. The use of fractioned regimens, rather than single fraction treatment, will likely improve outcomes given the inferior outcomes seen with single fraction HDR-BT brachytherapy in the whole-gland setting.^43^ Given the predominantly marginal and out-of-field recurrence seen with focal brachytherapy, intensification of treatment with treating elective prostate volumes (with a dose painting approach) or with conventional systemic therapies such as androgen deprivation therapy or novel agents may further improve outcomes, although these approaches could be associated with increased toxicity.

Although there is significant heterogeneity in time intervals of BC in the studies included for this meta-analysis, we consider supporting the use of F-BT as monotherapy with the further development of prospective clinical trials comparing this strategy versus whole-gland treatment and its inclusion and technical detailing in appropriate clinical guidelines as a valid therapeutic approach for definitive treatment in localized PCa.

### Limitations

As with all meta-analysis, the study could not take into account the possibility of selective reporting of outcomes of each of the studies included as the BC rates were reported at varying time points and intervals which might affect the validity of results and the ability to compare and aggregate results properly.^52^ As well, there was heterogeneity of *I*^2^ > 50% in four of 12 analyses, leading to the use of random effects estimates. While there is notable heterogeneity for some analyses, it may be expected that meta-analyses of proportions tend to have greater *I*^2^ estimates and therefore concerns of heterogeneity,^53^ which may also be influenced by the variability in modality and dose fractionation. Moreover, there was variability in the reporting of toxicities with some studies reporting in CTCAE and other reporting in RTOG toxicity scales with a number of studies not reporting toxicities at all and quality of life was also only reported in nine out of the 26 studies with the studies using varying scales to determine QoL. Additionally, there is a lack of long-term toxicity data which is particularly relevant in the salvage brachytherapy cohort.

The quality of each of the studies in the analysis was also assessed showing that the majority of studies, 16 (72.8%), demonstrated a low risk of bias, while six studies (22.2%) exhibited a moderate risk of bias. Of the 26 studies included, six were retrospective studies, which might introduce selection and memory bias into the aggregated data, and there was a lack of randomized phase 3 trials, which are considered to provide the highest-quality data for meta-analyses.

Another limitation is the heterogeneity in techniques with a variability in the dosing regimen, modality with some utilizing LDR and others utilizing HDR brachytherapy as well the technique for target identification and delineation with the majority (88%), but not all, utilizing MRI with or without biopsy and PSMA PET-CT scan. The variability in the imaging modality used for target delineation could confer differences in the adequacy of tumour coverage.

In terms of the target treated itself, 15 of the studies treated the GTV plus a 1-7 mm margin, whereas five of the studies included treated the hemigland or the peripheral zone only which has ramifications as partial gland or hemigland treatment would entail treating a larger volume of the prostate. As the patients were not included *via* random sampling the results cannot be used to determine causality, but the results would still be able to provide a glimpse of the general outcomes of patients receiving focal or partial gland brachytherapy to the prostate in both the monotherapy and salvage settings.

## Conclusion

As prostate cancer outcomes continue to improve, there has been increasing emphasis on methods to improve quality of life and focal brachytherapy appears to be an attractive option. Our results show that focal brachytherapy appears to have a favourable toxicity profile with encouraging 5-year BC rates, albeit lower than whole-gland treatment approaches on comparison with historical data. The studies that reported on patterns of recurrence have shown that most local recurrences are marginal or outside of the treated volume, and both clinicians and patients should be aware of this finding during decision-making.

The authors recommend future trials to determine the best possible interplay between focal techniques such as F-BT together with whole-gland techniques such as possible attenuation of whole-gland dose and escalating doses to the DIL as well as more investigations on multi-parametric MRIs and PSMA PET-CT scans to more accurately determine the actual extent of disease to further refine and improve outcomes in F-BT.

## Supplementary Material

tqae254_Supplementary_Data
