# Supplementary material for: Partial or focal brachytherapy for prostate cancer: a systematic review and meta-analysis
Source: Br J Radiol. 2024 Dec 19;98(1167):354–67. doi: 10.1093/bjr/tqae254 (PMC11840170; doi:10.1093/bjr/tqae254)

Supplementary 3. Risk of Bias

Figure A. Overall risk of bias assessment as per ROBINS I tool.

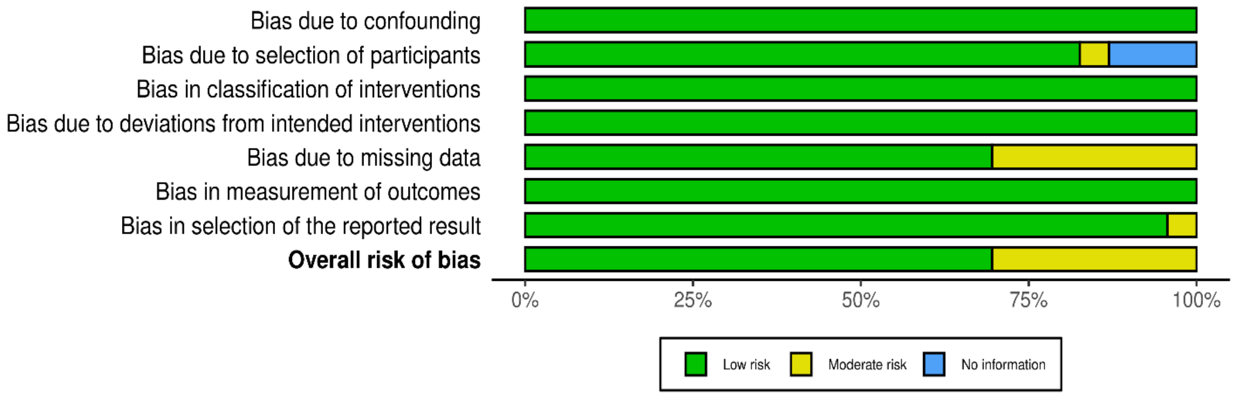

Figure B. Risk of bias assessment by ndividual studies according to ROBINS I tool.

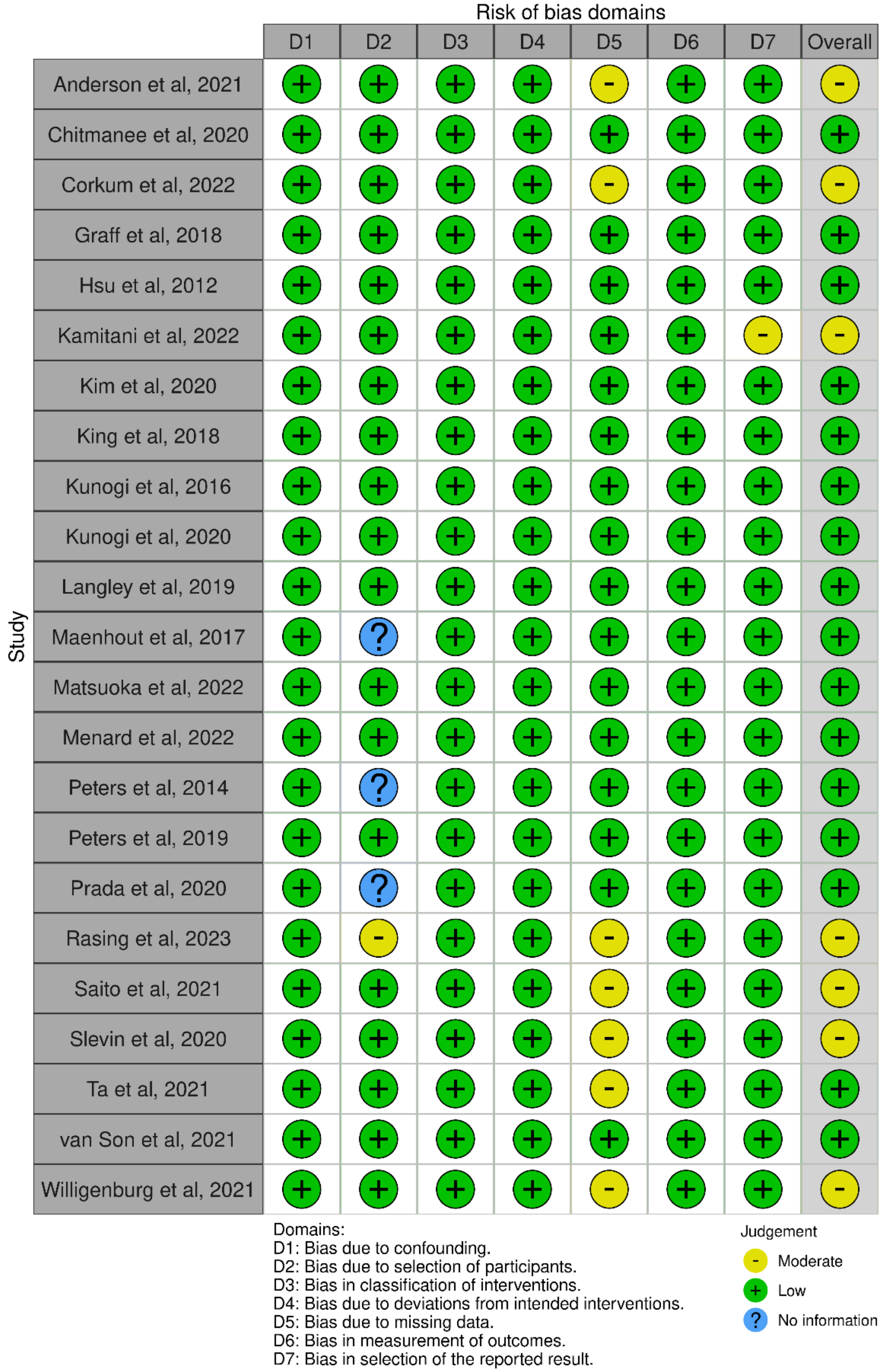

Supplement: tqae254_Supplementary_Data [file tqae254_supplementary_data.zip › tqae254_Supplementary_Data/SUPPLEMENTARY 3.pdf]
