# Supplementary material for: Partial or focal brachytherapy for prostate cancer: a systematic review and meta-analysis
Source: Br J Radiol. 2024 Dec 19;98(1167):354–67. doi: 10.1093/bjr/tqae254 (PMC11840170; doi:10.1093/bjr/tqae254)
Supplement: tqae254_Supplementary_Data [file tqae254_supplementary_data.zip › tqae254_Supplementary_Data/Supplementary1 (1).docx]

**SUPPLEMENTARY 1a.** Search Strategy

**PRISMA Chart Numbers:**

| Database | Total retrieved |
| --- | --- |
| Medline ALL 1946 to July 05, 2023 | 5403 |
| Embase Classic +Embase 1947 to 2023 July 05 | 8018 |
| Cochrane Central Register of Controlled Trial June 2023 | 253 |
| Cochrane Database of Systematic Reviews July 5, 2023 | 5 |
| Web of Science July 7, 2023 | 2104 |
| SubTotal | 15783 |

**SUPPLEMENTARY 1b.** Search Strategy

**Ovid MEDLINE(R) ALL 1946 to July, 2023**

|  |  |  |  |  |  |  |
| --- | --- | --- | --- | --- | --- | --- |
| **#** | **Searches** | **Results** | **Type** |  |  |  |
|  | | | | | | |
| 1 | exp Prostatic Neoplasms/ | 143176 | Advanced |  |  |  |
| 2 | Prostate-Specific Antigen/ | 29629 | Advanced |  |  |  |
| 3 | Prostate/ | 41543 | Advanced |  |  |  |
| 4 | (prostat* adj6 neoplas*).mp,kw. | 151229 | Advanced |  |  |  |
| 5 | (prostat* adj6 cancer*).mp,kw. | 160408 | Advanced |  |  |  |
| 6 | (prostat* adj6 tumo?r*).mp,kw. | 27878 | Advanced |  |  |  |
| 7 | (prostat* adj6 oligo*).mp,kw. | 1358 | Advanced |  |  |  |
| 8 | (prostat* adj6 malign*).mp,kw. | 7387 | Advanced |  |  |  |
| 9 | (prostat* adj6 carcin*).mp,kw. | 23689 | Advanced |  |  |  |
| 10 | (prostat* adj6 adenocarcin*).mp,kw. | 10669 | Advanced |  |  |  |
| 11 | (prostat* adj6 invad*).mp,kw. | 213 | Advanced |  |  |  |
| 12 | (prostat* adj6 invas*).mp,kw. | 3595 | Advanced |  |  |  |
| 13 | (prostat* adj6 residual*).mp,kw. | 1364 | Advanced |  |  |  |
| 14 | (prostat* adj6 (recur* or re-cur*)).mp,kw. | 9066 | Advanced |  |  |  |
| 15 | (prostat* adj6 (reoccur* or re-occur*)).mp,kw. | 2 | Advanced |  |  |  |
| 16 | (prostat* adj6 relaps*).mp,kw. | 1488 | Advanced |  |  |  |
| 17 | (prostat* adj6 onco*).mp,kw. | 4148 | Advanced |  |  |  |
| 18 | (prostat* adj6 castrat*).mp,kw. | 13611 | Advanced |  |  |  |
| 19 | CAPRA.mp,kw. | 2033 | Advanced |  |  |  |
| 20 | CaPSURE.mp,kw. | 222 | Advanced |  |  |  |
| 21 | or/1-20 | 228213 | Advanced |  |  |  |
| 22 | Brachytherapy/ | 21848 | Advanced |  |  |  |
| 23 | (brachy* adj5 focal*).mp,kw. | 145 | Advanced |  |  |  |
| 24 | (brachy* adj5 partial*).mp,kw. | 275 | Advanced |  |  |  |
| 25 | (brachy* adj5 salvag*).mp,kw. | 357 | Advanced |  |  |  |
| 26 | (focal* adj5 salvag*).mp,kw. | 177 | Advanced |  |  |  |
| 27 | (partial* adj5 salvag*).mp,kw. | 364 | Advanced |  |  |  |
| 28 | (brachy* adj5 boost*).mp,kw. | 962 | Advanced |  |  |  |
| 29 | (focal* adj5 boost*).mp,kw. | 141 | Advanced |  |  |  |
| 30 | (partial* adj5 boost*).mp,kw. | 220 | Advanced |  |  |  |
| 31 | (salvag* adj5 boost*).mp,kw. | 53 | Advanced |  |  |  |
| 32 | (brachy* adj5 high-dose*).mp,kw. | 4071 | Advanced |  |  |  |
| 33 | (focal* adj5 high-dose*).mp,kw. | 135 | Advanced |  |  |  |
| 34 | (partial* adj5 high-dose*).mp,kw. | 450 | Advanced |  |  |  |
| 35 | (salvag* adj5 high-dose*).mp,kw. | 579 | Advanced |  |  |  |
| 36 | (brachy* adj5 HDR).mp,kw. | 2702 | Advanced |  |  |  |
| 37 | (focal* adj5 HDR).mp,kw. | 25 | Advanced |  |  |  |
| 38 | (partial* adj5 HDR).mp,kw. | 39 | Advanced |  |  |  |
| 39 | (salvag* adj5 HDR).mp,kw. | 65 | Advanced |  |  |  |
| 40 | (brachy* adj5 low-dose*).mp,kw. | 1330 | Advanced |  |  |  |
| 41 | (focal* adj5 low-dose*).mp,kw. | 62 | Advanced |  |  |  |
| 42 | (partial* adj5 low-dose*).mp,kw. | 327 | Advanced |  |  |  |
| 43 | (salvag* adj5 low-dose*).mp,kw. | 86 | Advanced |  |  |  |
| 44 | (brachy* adj5 LDR).mp,kw. | 662 | Advanced |  |  |  |
| 45 | (focal* adj5 LDR).mp,kw. | 10 | Advanced |  |  |  |
| 46 | (partial* adj5 LDR).mp,kw. | 3 | Advanced |  |  |  |
| 47 | (salvag* adj5 LDR).mp,kw. | 15 | Advanced |  |  |  |
| 48 | HDR-BT.mp,kw. | 431 | Advanced |  |  |  |
| 49 | LDR-BT.mp,kw. | 118 | Advanced |  |  |  |
| 50 | CLDR-BT.mp,kw. | 1 | Advanced |  |  |  |
| 51 | curietherap*.mp,kw. | 841 | Advanced |  |  |  |
| 52 | curie-therap*.mp,kw. | 42 | Advanced |  |  |  |
| 53 | or/22-52 | 26151 | Advanced |  |  |  |
| 54 | 21 and 53 | 5874 | Advanced |  |  |  |
| 55 | exp animal/ not (exp animals/ and exp humans/) | 5136819 | Advanced |  |  |  |
| 56 | 54 not 55 | 5852 | Advanced |  |  |  |
| 57 | limit 56 to english language | 5403 | Advanced |  |  |  |
|  | | |  |  |  |  |
