# Supplementary material for: Partial or focal brachytherapy for prostate cancer: a systematic review and meta-analysis
Source: Br J Radiol. 2024 Dec 19;98(1167):354–67. doi: 10.1093/bjr/tqae254 (PMC11840170; doi:10.1093/bjr/tqae254)
Supplement: tqae254_Supplementary_Data [file tqae254_supplementary_data.zip › tqae254_Supplementary_Data/Supp_2_Aug_23_Biochemica control definition.docx]

Biochemical control (BC) was defined as the absence of biochemical failure. The specific definitions used in each study are presented below

**Supplementary 2 A and 2 B**

| Study | Biochemical Failure Definition |
| --- | --- |
| Graff 2018 | Biochemical relapse (not reported). |
| King 2018 | Phoenix criterion (PSA nadir + 2 ng/mL) plus a PSA velocity ≥0.75 ng/mL per year between the nadir and the value before salvage. |
| Maenhout 2018 | Phoenix criterion (PSA nadir + 2 ng/mL). |
| Langley 2019 | Biochemical failure (not specified). |
| Peters 2019 | Phoenix criterion (PSA nadir + 2 ng/mL). |
| Kim 2020 | Phoenix criterion (PSA nadir + 2 ng/mL). |
| Kunogi 2020 | Phoenix criterion (PSA nadir + 2 ng/mL). |
| Prada 2020 | Phoenix criterion (PSA nadir + 2 ng/mL). |
| Anderson 2021 | Phoenix criterion (PSA nadir + 2 ng/mL). |
| Saito 2021 | Phoenix criterion (PSA nadir + 2 ng/mL). |
| Ta 2021 | Phoenix criterion (PSA nadir + 2 ng/mL). |
| Matsuoka 2022 | Phoenix criterion (PSA nadir + 2 ng/mL). |

1. **Monotherapy studies included**

| Study | Biochemical Failure Definition |
| --- | --- |
| Hsu 2012 | Phoenix criterion (PSA nadir + 2 ng/mL) or the ASTRO consensus definition (3 consecutive rising PSA measurements, with the failure date as the midpoint between the first rise and the previous measurement), or biopsy-proven recurrence. |
| Peters 2014 | Phoenix criterion (PSA nadir + 2 ng/mL). |
| Kunogi 2016 | Phoenix criterion (PSA nadir + 2 ng/mL). |
| Maenhout 2017 | Phoenix criterion (PSA nadir + 2 ng/mL). |
| Chitmanee 2020 | Phoenix criterion (PSA nadir + 2 ng/mL). |
| Slevin 2020 | Phoenix criterion (PSA nadir + 2 ng/mL). |
| Willigenburg/Van Son 2021 | Phoenix criterion (PSA nadir + 2 ng/mL). |
| Corkum 2022 | Phoenix criterion (PSA nadir + 2 ng/mL). |
| Menard 2022 | Phoenix criterion (PSA nadir + 2 ng/mL). |
| Rasing 2023 | Phoenix criterion (PSA nadir + 2 ng/mL). |

1. **Salvage studies included**
